# Experiences and rehabilitation needs of runners with anterior knee pain in under-resourced communities in Ekurhuleni, Gauteng, South Africa

**DOI:** 10.17159/2078-516X/2020/v32i1a6969

**Published:** 2020-01-01

**Authors:** S H Kunene, N P Taukobong, S Ramklass

**Affiliations:** 1 Physiotherapy Department, University of the Witwatersrand, Johannesburg, South Africa; 2 Institutional Planning Department, Sefako Makgatho Health Sciences University, Pretoria, South Africa; 3 School of Clinical Medicine, University of KwaZulu-Natal, Durban, South Africa

**Keywords:** patellofemoral pain syndrome, encounters, health needs, running, poorly-resourced communities

## Abstract

**Background:**

Anterior knee pain (AKP) is a common knee injury resulting from overuse, and impact negatively on the quality of life of many runners. Runners with AKP in under-resourced poor communities present with poor health outcomes.

**Aim:**

To determine the experiences and rehabilitation needs of runners in under-resourced communities in Ekurhuleni, South Africa.

**Methods:**

The study was qualitative, based on the focus group interview method. Interviews were conducted with 12 runners. They were aged from 18 to 45 years and had a history of AKP. Permission was obtained from club managers and consent from each participant. An interview schedule with predetermined questions was used to collect the data. Two researchers conducted the interview, a facilitator and moderator. The interview session lasted for 80 minutes. Audio recordings of the interview session were made, transcribed verbatim and notes taken, with the final result provided in a written report. The data approach was thematic and deductive in nature.

**Results:**

All 12 recruited participants participated. The participants were comprised of six females and six males, eight youths and four adults; seven had ≤5 years of running experience and five had 10 years. The following themes and subthemes emerged: 1) The negative impact of AKP on health (physical, emotional and social); 2) Limited rehabilitation services (availability, accessibility, affordability, adequacy and appropriateness); 3) Rehabilitation needs (knowledge and professional intervention).

**Conclusion:**

The study showed the negative impact of AKP on health and the problem of the paucity of rehabilitation services. A community based rehabilitation programme is therefore recommended for runners.

Anterior knee pain (AKP) has a negative impact on the quality of life (QOL) of many athletes,^[1,2]^ affecting their physical, psychosocial, emotional and mental health. This condition is most common among runners, accounting for between 15–45% of the cases of AKP globally.^[3]^ In a previous study, Kunene et al.^[4]^ found that 40% of the runners in under-resourced communities in Ekurhuleni, South Africa, presented with AKP. This condition is was found to be more prevalent in females, the youth, and runners with a limited number of years of running experience (less than five years).^[3]^

Various factors were found to contribute to this condition. According to Kunene et al.^[4]^ tight hamstring and iliotibial band muscles, weak quadriceps muscles, weak gluteal muscles (resulting in poor hip control) and patellar tilt problems, were found to contribute significantly to AKP. Other factors may include tight iliopsoas muscles, tightness of the gastrocnemius complex, foot pronation, general joint laxity, leg length discrepancy, patellar glide abnormalities, and patellar hypermobility.^[4,5]^ Kunene et al.^[5]^ also found that a heavy training load, lack of warm-up exercises during training, poor condition of running shoes, and downhill running significantly contribute to AKP among runners.^[6]^

The incidence of sports injuries and their associated problems continues to increase among athletes, more especially among those in the poorer communities who live under unfavourable socioeconomic conditions.^[7]^ These factors may include paucity of health services, limited access to coaching professionals, and sports administrators available to organise and deliver sports events, and negative attitudes towards sports-related injuries.^[7]^ The lack of health services could be as a result of no formal health structures with their associated support facilities and of trained professionals to deliver these services. The lack of health resources is a serious challenge in many African countries, including South Africa.^[8]^ Most health facilities in the rural and peri-urban communities do not have access to full rehabilitation teams. Therefore, athletes do not receive the necessary holistic treatment that they require for their injuries. While it is ideal to have access to full rehabilitation teams and the services they provide in sporting clubs, owing to their financial constraints, poorer communities do not have access to these facilities. Thus many athletes end up training, and even competing, with their untreated injuries, which in turn lead to further complications. In fact, many runners may terminate their running careers due to injury complications.

In professional sports, especially in urban communities, athletes are fortunate to have access to specialised sports rehabilitation services offered either by their clubs, or privately. In addition to the availability and accessibility of these rehabilitation services, there are also personal trainers and coaches for athletes in urban communities. Unfortunately, this is not so in the poorer rural and peri-urban communities.

In the course of this research, which was conducted at running clubs located in communities with low socioeconomic status, it was noted that some runners had stopped running as a result of their injuries, while others were running with injuries that had not been addressed. There were no rehabilitation services for injured sportspeople in these communities.

In order to design appropriate rehabilitation solutions for these runners, it was necessary to fully understand their experiences and rehabilitation needs. The purpose of this study was intended to determine the experiences and rehabilitation needs of runners in under- resourced communities in Ekurhuleni, South Africa.

## Methods

### Study design

This study was a qualitative study involving focus group interviews. The focus group interview was used to trigger responses from participants in order to clarify their experiences and rehabilitation needs.

### Participants

The twelve participating runners included long-distance, cross country, and those with a history of AKP as determined by a qualified physiotherapist. These were the runners who participated in these authors’ previous studies^[2,4,6]^ In these previous studies, the prevalence of AKP was determined by the use of the Kujala AKP questionnaire (a subjective tool) and confirmed by objective screening tests^[2,4]^. Participants were members of six developing running clubs in under-resourced communities in Ekurhuleni, South Africa. Runners with degenerative conditions and traumatic injuries were excluded. The interviews took place in a quiet office at one of the training facilities.

### Recruitment

Ethical clearance to conduct this study was obtained from the University of KwaZulu-Natal (clearance number is BFC377/15) and permission to conduct the study was given in writing by the respective club managers. A letter of invitation was sent to each club manager from the six developing running clubs requesting the participation of at least two of their runners who were a minimum of 18 years old and in training, who had undergone physical screening for AKP in the authors’ previous studies.^[2,4]^. The letter of invitation included information on the proposed date, time and venue for the interview.

### Data collection

A focus group interview was scheduled by the researchers to collect detailed data from the participants with regard to their running experiences and rehabilitation. It was developed by the first author and validated for content by three physiotherapy experts with sports clinical and research experience. The schedule included predetermined questions about the participants’ experiences (injury and rehabilitation) and their rehabilitation needs. A pilot study was conducted to test the appropriateness of the data collection tool prior to the main study.

The focus group discussion was facilitated by the first author (interviewer) and a research assistant, who presided over the process and also took care of the administrative aspects, which included the taking of notes and ensuring that all of relevant documentation was in order, e.g. information sheets, consent forms, interview notes etc. The discussion was undertaken using all 12 participants in one meeting. On the first day of data collection, the main author provided a statement of the purpose of the interview and allowed the participants to go through the study’s information sheet. All of the participants were requested to sign the consent form, and they also gave their permission for the interview to be audio recorded.

The first author outlined the procedures of the meeting and encouraged everyone to participate in the discussion. The interview was conducted in English as it was the language best understood by all the participants. The first author then posed the questions as predetermined in the schedule. Follow-up probe questions were also used to garner in-depth insights into the information pertaining to the main question asked. Participants were encouraged to answer the questions and express their experiences freely, rather than to find consensus. The questions covered the following topics: injury experiences, rehabilitation experiences, and rehabilitation needs. The session lasted for 80 minutes. The audio recordings were transcribed verbatim and handwritten notes were taken.

### Statistical analysis

The names of the participants were coded numerically in the transcripts. The data were analysed thematically, the process being deductive in nature. The first author and research assistant reviewed the transcripts independently. They then compared their findings to reach agreement on the themes and subthemes from the data. The process included the following steps: 1) familiarisation with the data by reading and re-reading the original transcript, while listening to the audio recording, 2) development of themes and subthemes from the concepts and categories from the data, and 3) defining and naming the themes and subthemes. Three rounds of discussions took place between the first author and the research assistant, where they compared the findings from the interviews to improve the credibility of the process.

## Results

All of the 12 participants took part in the focus group interview meeting. The participants included six females and six males: eight youths (18–35 years) and four adults (36–45 years). Seven of the participants had less than five years of running experience and five had between five and ten years of running experience (Table 1).

The participants’ injury and rehabilitation experiences, and rehabilitation needs are presented below, according to the selected themes and subthemes, together with their comments. The themes and subthemes are presented in Table 2.

### Experiences

#### Negative impact of AKP on health

All of the participants reported that they had experienced AKP injuries in the past as a result of their long-distance running, and that they were still suffering from these injuries. Their injuries had been confirmed by a qualified clinician. According to their descriptions, their pain was located on the anterior aspect of the knee below the patella. They received these injuries during training and exacerbated during races. These injuries negatively affected their health.

##### Physical impact

The participants expressed their frustrations with knee pain when running. They indicated that the pain interferes with their running to the point that they are being forced to miss training sessions and races. *‘I have had bad experiences in the past, and now too, with my knee problems. My pain has affected the way I run. In the beginning of the training or race I feel the pain, but as time goes on, the pain disappears but also comes back later as I run especially when I feel I’m getting tired (male, 38).’ ‘I also have the same challenge as my brother. Mine is worse during training, especially on an uneven surface, especially here on our training ground’ (male, 30)*

##### Emotional impact

Some indicated that their condition negatively impacted not only on their physical health, but also on their emotional health. Some reported that they sometimes become stressed about their situation and ended up being demotivated and not wanting to continue with running. *‘I sometimes get stressed when I think of how my knee problems have affected my performance as a runner, because running is my life’ (male, 36)*.

##### Social impact

All participants agreed that running is a social activity that keeps them connected with people, especially their friends. Some use running as a means to improve their physical appearance so that they are accepted among their peers. Therefore, sustaining injuries threatens not only the physical and emotional aspects of their life, but also their social life. *‘Running is our way of life, it gives some of us the purpose for living. So, this knee problem I have really makes me very emotional and it takes away my sense of belonging, especially when I think of the possibility of being unable to run anymore because of it’ (female, 28)*.

##### Poor experiences with rehabilitation services

#### Availability

Participants reported that their communities do not have rehabilitation services to address their needs as is the case in more affluent urban communities. They indicated that the clinics in their vicinity only provide basic medical services but not rehabilitation programmes for sports-related injuries. *‘This place does not have a clinic or somebody who is helping us with the injury problems we are facing. The only thing you can get is pain tablets when you go to the clinic. They don’t even give you a bandage’ (female, 31)*. Runners end up trying to find their own solutions to the injury problems they are facing. *‘What I’m using currently is Arnica oil. I sometimes ask someone to massage my knee and thigh muscle using Arnica oil. I sometimes use ice baths or rub my knee with ice. When I wake up the following day, I feel better. When I don’t massage, I feel stiff and have more pain’ (female, 20)*.

##### Accessibility

Participants indicated that rehabilitation services can only be found at the district or regional hospitals, which are located far from their communities. Sometimes even the hospitals have limited services. Accessing these services is a long process. Participants reported that an injured person has to first visit the clinic in order to get a referral to a hospital in order to access the rehabilitation services offered there. When they finally get to the hospital, the athletes are required to stand in long queues. Sometimes athletes reach the hospital but do not immediately gain access to rehabilitation services. They are sometimes given an appointment for another day. *‘This thing of hospital, sometimes I don’t see a point of even trying to go there because it’s a too long process’ (male, 40)*. Most participants reported that the means of transport to the hospital for rehabilitation services is scarce. Sometimes, because of the distance, they are required to take more than one taxi.

##### Affordability

Participants reported that most of the services at the public clinics and hospitals are free of charge, especially for people who are not working or who fall into the lower income bracket. Most participants agreed that private practitioners where too expensive for them which is why they do not even consider visiting them. *‘No, those people (private practitioners) are too expensive for people like us who do not have enough money. It is better to deal with your problems yourself than to lose money like that’ (female, 34)*. Transport to clinics and hospitals is another factor that makes such services unaffordable to participants *‘I’m currently not working, and I don’t have money for transport to go to hospital and that is why I end up not honouring my appointments’ (male, 40)*.

##### Adequacy

The majority were of the opinion that the rehabilitation services at the hospitals and clinics are unable to prevent injuries among the running population or to offer the necessary treatment. They indicated that the rehabilitation services offered by their sporting clubs are very limited – few and far between, as opposed to those offered by the professional athletic clubs in more affluent urban communities. *‘Big clubs are better than us because they have their own physiotherapists or doctors to take care of them, but us we don’t have anyone because of money issues’ (male, 36)*.

##### Appropriateness

A few of the participants, especially those who could afford rehabilitation services, were positive about the standard of the services provided to them in hospitals and private practices. *‘I would say yes, when you eventually get to see a physio in a public hospital, you get the help you need. But I still prefer services in private’ (female, 19)*. They complained that the available healthcare centres do not have specialised practitioners to deal with running-related injuries (e.g. sports physicians, sports therapists).

### Rehabilitation needs

#### Knowledge

The interviews revealed that participants needed to be provided with the appropriate information regarding their injuries and how to manage them. *‘Maybe if we can get some material with information so that we can help ourselves. In that way we would not need to stress a lot and go very far to get help’ (female, 29). ‘We would like to have someone to advise us on the kind of shoes we need to use when we run because I think my problem with my knee is caused by the shoes I’m using’ (male, 45)*.

#### Intervention

Participants agreed that they need the trained people in their clubs to intervene. Such an intervention would include the frequent screening of injuries; supplying information on prevention tactics to avoid injury, and providing treatment for and rehabilitation techniques to deal with, the injury. ‘*Like in other big clubs, we also need help in terms of screening and treatment of injuries’ (male, 25). ‘I would go straight to the point. I wish our club to can have a physiotherapist who can help us with screening and treatment of injuries. Even if that physio come once in a while, it’s still fine. It’s unfortunate that there are not physiotherapists who can volunteer to come and help us’ (male, 42)*. Another intervention that they would like was the support of a person with coaching skills who could help them to improve their running performance. *‘We need trained coaches who will make us perform better, like other athletes in big countries’ (male, 22)*. Other participants also indicated that their training facilities were not in a good state of repair and that they would like them to be improved. *‘If government can help us improve our running facilities..., we are struggling, our running tracks are not good and the grounds are not levelled’ (male, 36)*.

## Discussion

The findings of this study highlighted the negative health impacts of AKP on participants, namely, the physical, emotional and social aspects. These findings are similar to those found in the authors’ previous survey on the impact of AKP on the quality of life among 73 runners.^[2]^ Maclachlan et al. also reported extensively on the impact of AKP on the psychological well-being of runners.^[9]^ They found that runners experienced stress, anxiety, depression, and fear of movement because of AKP. This suggests a need to diligently deal with AKP and its associated problems. Thus a comprehensive rehabilitation programme is required to address both the physical and non-physical features of this condition in order to improve runners’ quality of life.

The study revealed that rehabilitation services are limited for the running population in the under-resourced communities of Ekurhuleni. Problems with availability, accessibility, affordability, adequacy, and appropriateness of rehabilitation services were reported. Furthermore, a lack of resources and limited skills, including health professional skills, is a serious challenge in South Africa, ^[8]^ particularly in socio-economically challenged communities. A low level of education, a problematical economy, and issues of crime, are some of the factors that contribute to this problem in South Africa.

People who live in poor communities have been found to present with poor mental and physical health outcomes.^[7]^ As previously mentioned, a lack of resources and limited professional skills available to them, could be some of the main reasons for limiting their well-being. Thus the prevalence of anterior knee pain was reported to be greatest among runners in under-resourced communities, such as those in the Ekurhuleni communities.^[4]^

This study’s results highlighted the need to address the problem of limited rehabilitation services for the running population in under-resourced communities. A need to empower runners with the relevant knowledge and skills for self-help and a need for professional intervention were identified. According to Esculier et al., educating runners about the symptoms of AKP and how to manage this condition is found to be effective in dealing with this type of injury.^[10]^ However, they still need the services and support from trained professionals. Rehabilitation services should be made available, accessible, affordable, appropriate and adequate for athletes in rural areas and in township communities. Healthcare resources need to be equally allocated to both the more affluent in the urban areas and the disadvantaged, the latter being burdened with the additional challenges of living at a lower socioeconomic level.

Rehabilitation teams in the professional sports arena, including various practitioners such as sports physicians, physiotherapists, psychologists, nutritionists or dieticians, biokineticists, massage therapists, podiatrists and other healthcare practitioners, depend on the needs of the injured person. Furthermore, in addition to these healthcare practitioners, personal trainers and coaches/managers also form part of the rehabilitation team. Each member of the team uses his/her knowledge and skills appropriately for each phase of rehabilitation. Communication and cooperation between the rehabilitation team members is vital in ensuring the continuity of care for runners. While it is ideal to have full rehabilitation teams to provide services for running clubs, it is not always possible to provide them in under-resourced communities. Therefore, the transdisciplinary rehabilitation model may be the solution to issues of limited professional healthcare services in under-resourced communities. This model is client-centred and promotes an ethos of cooperation among the rehabilitation team members to provide worthwhile rehabilitation services. With this approach, the rehabilitation team members are cross-trained and multi-skilled in several areas besides in their own speciality, in order to provide a holistic rehabilitation service.^[11]^

Other studies have provided convincing evidence about the importance and effectiveness of the transdisciplinary approach.^[12,13,14,15]^ For this model to work effectively, rehabilitation team members are encouraged to consult with one another, to learn to be interdependent, to assume interchangeable roles and responsibilities, and to exchange information, knowledge and skills.^[15]^

### Limitations

The study presented with some limitations. The researcher could not draw participants from a larger population due to restricted resources, thus the findings from the interviews cannot be applied more widely. Runners younger than 17 years and older than 45 years were not included in the study. Further studies may be necessary to include a larger, more diverse and more dispersed population to better understand the experiences of runners of various ages, gender and running experiences, and found in different environments.

## Conclusion

Anterior knee pain is a common but serious injury to the knee that is experienced by many runners. This study revealed that AKP negatively impacts on the physical, emotional and social health of runners in under-resourced communities. The study also showed that rehabilitation services are limited in terms of their availability, accessibility, affordability, adequacy and appropriateness, and therefore in the management of running-related injuries in these communities.

Issues such as the need to push for the empowerment of runners through self-help knowledge and skills, as well as to bring them to the realisation that there is a need for professional intervention in order to manage their injuries, were identified. Therefore, a transdisciplinary rehabilitation approach is recommended as an intervention strategy to cater for the needs of runners in under-resourced communities.

## Figures and Tables

**Table 1:** Characteristics of participants

Characteristics	n (%)
**Gender**	
Male	6 (50)
Female	6 (50)
**Age (y)**	
18–35	8 (67)
36–45	4 (33)
**Years of running**	
<5	7 (58)
5–10	5 (42)
>10	0

**Table 2:** Participant themes and subthemes

	Themes	Subthemes
**Injury and rehabilitation experiences**	**Negative impact of AKP on health**	Physical impact
		Emotional impact
		Social impact
	**Poor experiences with rehabilitation services**	Unavailability of services
		Inaccessibility of services
		Unaffordability of services
		Inadequacy of services
		Inappropriateness of services

**Rehabilitation needs**	**Knowledge**	About their condition
		About self-management
	**Intervention**	Need for injury screening
		Need for professionals for provide services
		Need for skilled coaching

AKP, Anterior knee pain

## References

[1] Cheung RT Zhang Z Ngai SP Different relationships between the level of patellofemoral pain and quality of life in professional and amateur athletes PMR 2013 5 7 568 572 10.1016/j.pmrj.2012.12.007 23375635

[2] Kunene SH Ramklass S Taukobong NP The impact of anterior knee pain on the quality of life among runners in under-resourced communities in Ekurhuleni, Gauteng S Afr J Sports Med 2018 30 1 6 10.17159/2078-516X/2018/v30i1a4947

[3] Cook C Hegedus E Hawkins R Diagnostic accuracy and association to disability of clinical test findings associated with patellofemoral pain syndrome Physiother Can 2010 62 1 17 24 10.3138/physio.62.1.17 21197175 PMC2841549

[4] Kunene SH Ramklass S Taukobong NP Anterior knee pain and its intrinsic risk factors among runners in under-resourced communities in Ekurhuleni, Gauteng S Afr J Physiother 2018 74 1 a452 10.4102/sajp.v74i1.452 PMC6191677 30349876

[5] Halabchi F Mazaheri R Seif-Barghi T Patellofemoral pain syndrome and modifiable intrinsic risk factors; how to assess and address? Asian J Sports Med 2013 4 2 85 100 10.5812/asjsm.34488 23802050 PMC3690728

[6] Kunene SH Ramklass S Taukobong NP Anterior knee pain and its extrinsic risk factors among runners in under-resourced communities in Ekurhuleni, Gauteng S Afr J Sports Med 2019 31 1 6 10.17159/2078-516X/2019/v31i1a6090 PMC9924583 36817999

[7] Finch C Mahoney M Townsend M Rural sports and recreational injuries in Australia: what do we know? Aust J Rural Health 2003 11 3 151 158 10.1046/j.1440-1584.2003.00500.x 12950399

[8] Rasool F Botha CJ The nature, extent and effect of skills shortages on skills migration in South Africa SA J Hum Resour Manag 2011 9 1 a287 10.4102/sajhrm.v9i1.287

[9] Maclachlan LR Collins NJ Matthews ML The psychological features of patellofemoral pain: a systematic review Br J Sports Med 2017 51 9 732 742 10.1136/bjsports2016-096705 28320733

[10] Esculier JF Bouyer LJ Dubois B Is combining gait retraining or an exercise programme with education better than education alone in treating runners with patellofemoral pain? A randomised clinical trial Br J Sports Med 2018 52 10 659 666 10.1136/bjsports-2016-096988 28476901

[11] Behm J Gray N Mauk K Kirsten L Interdisciplinary rehabilitation team Rehabilitation nursing: a contemporary approach to practice Sudbury, MA Jones & Bartlett Learning 2012 51 62

[12] Moodley L Louw B Hugo R Early identification of at-risk infants and toddlers: a transdisciplinary model of service delivery S Afr J Commun Disord 2000 47 25 39 11455820

[13] Reilly C Transdisciplinary approach: an atypical strategy for improving outcomes in rehabilitative and long-term acute care settings Rehabil Nurs 2001 26 6 216 220 10.1002/j.2048-7940.2001.tb01958.x 12035721

[14] Stepans MB Thompson CL Buchanan ML The role of the nurse on a transdisciplinary early intervention assessment team Public Health Nurs 2002 19 4 238 245 [ 10.1046/j.1525-1446.2002.19403.x 12071897

[15] King G Strachan D Tucker M The application of a transdisciplinary model for early intervention services Infants & Young Children 2009 22 3 211 223 10.1097/IYC.0b013e3181abe1c3

